# Evaluation of the Efficacy of a Neem Extract-Based Herbal Ointment, Herbal Antioxidant, and Propolis on Oxidative Stress Related to Oral Ulcers: An Interventional Animal Study

**DOI:** 10.7759/cureus.48542

**Published:** 2023-11-08

**Authors:** Aditya Patel, Shraddha A Patel, Joyeeta Mahapatra, Disha Mehta, Sabari Murugesan, Savadamoorthi Kamatchi Subramani

**Affiliations:** 1 Department of Conservative Dentistry and Endodontics, Sharad Pawar Dental College and Hospital, Wardha, IND; 2 Department of Oral Medicine and Radiology, Sharad Pawar Dental College and Hospital, Wardha, IND; 3 Department of Conservative Dentistry and Endodontics, College of Dentistry, Jazan University, Jazan, SAU

**Keywords:** neem extracts, propolis, wound contraction, antioxidant activity, hemidesmus indicus, oxidative stress, traumatic oral ulcers

## Abstract

Traumatic oral ulcers are one of the most commonly encountered oral ulcers. Their healing may be delayed due to factors like the presence of opportunistic infectious microbes in the oral cavity, secondary trauma from sharp edges of teeth, and the systemic condition of the patient.

Aim: To compare the efficacy of a newly developed neem extract containing herbal ointments (propolis and *Hemidesmus indicus)* in enhancing the wound contraction of traumatic oral ulcers and to determine the relationship between oxidative stress and oral ulcers.

Method: Ulcers were inflicted by trauma in the mouths of experimental rabbits using a 5 mm punch biopsy device. Forty-eight animals were randomly put into six groups (n = 12). Group 1 was the control group that did not receive any intervention; Group 2 had a systemic treatment of *Hemidesmus indicus* extract; Group 3 received a topical application of propolis; Group 4 had a topical application of a neem extract-based herbal ointment; Group 5 was administered a combination of *Hemidesmus indicus* and propolis; and Group 6 had a combination of a neem-based herbal ointment and *Hemidesmus indicus*. Oxidative stress levels were calculated by measuring superoxide dismutases and malondialdehyde levels in the blood on days 0, one, seven, and 14. Wound contraction scores of ulcers were also assessed on days seven and 14.

Results: Significantly higher wound contraction scores were seen in groups treated with herbal ointment in comparison to groups treated without herbal ointment. Oxidative stress levels increased in all groups after the infliction of ulcers (day one) and then declined as the ulcers healed, reaching near-normal levels on day 14. Groups containing *Hemidesmus indicus* showed a significant reduction in oxidative stress in comparison to groups without *Hemidesmus indicus*. A p-value of <0.05 was considered significant.

Conclusion: A combined formulation of herbal ointment and *Hemidesmus indicus* proved to be the most efficacious in enhancing wound contraction of oral ulcers along with significantly reducing oxidative stress in experimental rabbits.

## Introduction

Ulcers due to trauma in the mouth are one of the most commonly encountered oral ulcers, with a prevalence ranging from 3.5% to 15.6% in different surveys conducted across the world [1-6]. Most mucosal wounds take five to seven days to heal [7,8]. However, this may get delayed due to factors like the presence of opportunistic infectious microbes in the oral cavity, secondary trauma from the sharp cusp of the tooth, and the systemic condition of the patient [9]. For centuries, people of different tribes around the world have been using medicinal plants, certain herbs, and animal derivatives, including turmeric, neem (*Azardica indica*) derivatives, aloe vera, propolis, bee wax, etc., for treating wounds and ulcers. Recently, these products have garnered attention in the field of modern medicine, and research has shown them to be a promising potential source of remedy for the management and treatment of wounds [10].

Oxidative stress (OS) happens because of the disbalance between the production and neutralization of free radicals in the body and plays an essential part in the alteration of the healing process after injuries. Reactive oxygen species (ROS) are oxidative products formed within the body during various biochemical reactions and cellular functions. Their formation is neutralized by the production of antioxidants. The role of ROS in the case of burn wounds has been studied in the past [11,12]. There has been definitive evidence presented in the past establishing the correlation between oral ulcers and oxidative stress. However, research data demonstrating the role of systemic antioxidants in the promotion of the healing of oral ulcers is still scant.

Malondialdehyde (MDA) is an end-oxidative output of lipid peroxidation that acts as an indirect marker of relative oxygen species activity that can be measured from plasma [13,14]. Superoxide dismutases (SOD) are a group of antioxidant enzymes that play a role in catalyzing the dismutation of superoxide into O_^2^_ and H_2_O_2_, which offers an antioxidant effect in almost all cells that have had exposure to oxygen. The concentration of SOD in plasma can be used to measure the antioxidant status of an individual [14]. The ratio of MDA to SOD provides a stress index, which is used to correlate with the severity of oxidative stress [13,14].

Many naturally derived products have been claimed to have antioxidant effects and also claim to promote the healing of various wounds in the human body. Propolis is one such medicinal agent that is extracted from bee wax and is recommended to be used in the management of oral ulcers. It has anti-microbial, anti-inflammatory, and antioxidant properties [15]. An ointment derived from the herbs was developed by Pathak et al. in 2006 and contained extraction products of neem leaves, *Linum usitatissiumum L.* (linseed) oil, and resin of *Shorea robusta* (shaal), which proved to have better efficacy than silver sulphadiazine in enhancing the healing of burn ulcers in experimental rabbits [16]. This herbal ointment also proved to be an efficient therapy to enhance the quality of histopathological healing of ulcers due to trauma in the mouths of experimental rabbits.

*Hemidesmus indicus*, a commonly used antioxidant in the Ayurvedic system of medicine, is known to treat several free radical-mediated disease conditions [17,18]. It has been demonstrated recently that the use of *Hemidesmus indicus* promotes the quality of histopathological healing of oral wounds in experimental rabbits. However, whether its use has any effect on oxidative stress or clinical wound healing is yet to be established. Hence, a study was needed to evaluate and compare the antioxidant efficacy of a new herbal ointment and propolis with and without the combination of *Hemidesmus indicus* and their effect on enhancing clinical wound healing of traumatically inflicted ulcers in the mouth in experimental rabbits.

The objective of the study was to observe the wound contraction score of oral ulcers due to trauma and oxidative stress levels in the control group of experimental rabbits that did not receive any intervention. Evaluation of the wound contraction score of traumatic oral ulcers and oxidative stress levels was done on the experimental rabbit groups treated with orally administered *Hemidesmus indicus*, topically applied propolis, topically applied new herbal ointment, combined therapy of topical application of propolis and oral administration of *Hemidesmus indicus*, and combined therapy of topical application of herbal ointment and oral administration of *Hemidesmus indicus*, and the results were compared with each other.

## Materials and methods

Forty-eight healthy male and female rabbits were recruited for the research. On the basis of the findings of a pilot study, the sample size was calculated for the expected event rate between 15% and a protection fold of 70% using the formula stated by Wang and Bhakai [19]. According to the calculated outcome, the sample size was 11.52 per group. Thus, 12 rabbits were recruited per group (n = 12). Thus, a total of 72 healthy male and female rabbits aged six months to three years and weighing 2.5 kg to 3.5 kg were selected. Rabbits with pregnancies or diseases were excluded from the study. Animals were kept in different cages under standardized conditions of light, temperature, and humidity. An ad libitum diet of standard chow and water was administered to them. This study did not involve sacrificing the animals. No deaths or disabilities were reported in the animals on which the study was conducted. The overall effect of the experiment on the health of the experimental rabbits was monitored by measuring the overall weight loss of each animal.

Since this research was conducted on experimental animals, Animal Research: Reporting of In Vivo Experiments (ARRIVE) guidelines were followed while designing the research [20]. The study got its clearance from the Institutional Ethics Committee of Datta Meghe Institute of Medical Sciences (DMIMS) (Ref No. DMIMS (DU)/IEC/2012-12/144) on February 22, 2013. It also received clearance from the Institutional Animal Ethics Committee of DMIMS (Ref No. DMIMSDU/IAEC/2013-14/53).

Herbal formulations 

*Hemidesmus indicus* (root powder), *Linum usitatissimum*, and *Shorea robusta* were obtained from the “Rasa-shala” of Mahatma Gandhi Ayurvedic College, Hospital, and Research Centre, Salod, Wardha. The formulation had been authenticated by the department of Rasa-shala of Mahatma Gandhi Ayurvedic College, Hospital and Research Center, Salod.

This formulation was made in the central research laboratory (DMIMS) in a sterilized environment. Twenty grams of neem leaves were subjected to boiling in 500 ml of linseed oil until the color of the oil became greenish. It was then cooled to room temperature and was later triturated with the powdered resin of 100 g of *Shorea robusta*. The contents were rinsed a hundred times using distilled water to obtain the purified content of the new herbal formulation. The preparation was made according to the previous study by Pathak and Patel [16].

Traumatic oral ulcers were inflicted on the labial mucosa of each specimen with a 5 mm punch biopsy device under ketamine injection on day 0. The animals were later divided into six groups (n=12).

Group 1: No drug was given to the rabbits in the group (untreated control).

Group 2: This group was administered orally with a root extract of *Hemidesmus indicus* (an antioxidant), diluted in distilled water at 5 mg/kg of body weight, from days one to 14 daily, thrice a day at eight-hour intervals.

Group 3: Rabbits of this group were treated with a topically applied alcohol-free extract of propolis (YS Eco Bee Farms) from day one to day 14 daily, thrice a day at eight-hour intervals.

Group 4: Rabbits of this group were treated with topically applied new herbal ointment (containing extracts of neem, resin, and linseeds) from day one to day 14, thrice a day at eight-hour intervals.

Group 5: Rabbits were administered a combination therapy of *Hemidesmus indicus* and propolis as per the above-mentioned protocol.

Group VI: Rabbits were administered a combination therapy of *Hemidesmus indicus* and herbal ointment as per the above-mentioned protocol.

Measurement of oxidative stress

Blood samples were drawn from the central/marginal ear vein of the rabbits before the infliction of ulcers (day 0) and were collected in plain sterile bulbs and sterile ethylenediaminetetraacetic acid (EDTA) bulbs for the estimation of superoxide dismutase (by Marklund and Marklund method) [19] and the estimation of MDA by the thiobarbituric acid method, respectively [18].

Oxidative stress was measured by the estimation of SOD and MDA values for all samples in all groups.

The stress index was calculated as follows: [18].

Stress index = MDA / SOD

The test was then repeated on days one, seven, and 14 in all the groups using the same method as described above.

Monitoring of wound contraction was done by gauging the progressive changes. The raw wound area was traced on transparent paper on days seven and 14. The trace was then transferred to a 1 mm2 graph sheet, from which the wound surface area was calculated. From the calculated surface area, the percentage of wound contraction was calculated (taking the initial size of the wound as 100%) using the following equation [20]. Wound contraction (%) was calculated as [(Initial Wound Size - Specific Day Wound Size)/Initial Wound Size]х 100.

Statistical analysis involved calculating the mean wound contraction scores for days seven and 14, and the stress index was calculated for all groups on day 0, day one, day seven, and day 14. A comparison of the means of wound contraction scores and stress index scores of all treatment groups (Groups 2-4) with the control group (Group 1) was done using the Dunnet D Test. Multiple comparisons were done using the Tukey test to compare the wound contraction and stress index of all treatment groups with each other. Pearson’s coefficient was used to determine the correlation between the wound contraction percentage and the stress index. The software used for analysis was SPSS 17.0 (SPSS Inc., Chicago, IL), and a p-value of <0.05 was considered significant.

## Results

Stress index scores for all groups increased from day 0 to day one then decreased on day seven and further declined on day 14. The wound contraction scores improved for all the groups from day seven to day 14 (Table 1).

**Table 1:** Mean scores of stress index and wound contraction in Groups 1 to 6 SD: standard deviation

Day	Group	Stress index		Wound contraction	
		Mean	SD	Mean	SD
Day 0	Group 1	6.07	1.64	-	-
	Group 2	5.97	1.09	-	-
	Group 3	6.18	1.07	-	-
	Group 4	5.95	1.08	-	-
	Group 5	6.48	0.94	-	-
	Group 6	5.86	0.78	-	-
Day 1	Group 1	37.86	11.05	-	-
	Group 2	34.92	7.35	-	-
	Group 3	33.17	8.03	-	-
	Group 4	35.09	7.70	-	-
	Group 5	37.55	11.55	-	-
	​​​​​​​Group 6	34.52	10.09	-	-
Day 7	Group 1	17.40	5.06	55.09	3.95
	Group 2	11.56	2.31	65.66	3.67
	Group 3	15.92	3.72	54.97	4.67
	​​​​​​​Group 4	13.75	4.16	68.53	2.77
	​​​​​​​Group 5	12.32	3.70	66.55	3.20
	​​​​​​​Group 6	10.64	1.74	70.47	1.92
Day 14	Group 1	8.30	1.42	80.64	4.15
	Group 2	6.60	0.79	88.38	3.14
	Group 3	7.95	1.00	84.52	3.87
	Group 4	7.33	0.89	92.26	2.65
	​​​​​​​Group 5	7.05	1.16	89.19	2.11
	​​​​​​​Group 6	5.52	1.00	95.11	1.51

All treatment groups showed significantly higher wound contraction scores than the control group on day seven, except for Group 3. On day 14, the wound contraction was significantly higher for all treatment groups in comparison to the control group (Table 2).

**Table 2:** Comparison of means of wound contraction scores of Groups 2–6 with the control group (Group 1) using the Dunnet test S: significant; NS: not significant

	Group		Mean difference (I-J)	Standard error	p-value	95% Confidence interval	
						Lower bound	Upper bound
Day 7	Group 2	Group 1	10.56	1.42	0.0001, S	6.9	14.22
	Group 3		-0.12	1.42	1.000, NS	-3.78	3.53
	Group 4		13.43	1.42	0.0001, S	9.77	17.09
	Group 5		11.45	1.42	0.0001, S	7.79	15.11
	Group 6		15.37	1.42	0.0001, S	11.71	19.03
Day 14	Group 2	Group 1	7.73	1.24	0.0001, S	4.52	10.94
	Group 3		3.87	1.24	0.012, S	0.65	7.08
	Group 4		11.61	1.24	0.0001, S	8.4	14.82
	Group 5		8.54^*^	1.24	0.0001, S	5.33	11.75
	Group 5		14.46	1.24	0.0001, S	11.25	17.67

On day seven as well as on day 14, maximum wound contraction was observed in Group 6 (herbal ointment and *Hemidesmus indicus* combination), followed by Group 4 (herbal ointment alone), and the difference was statistically superior in comparison to the control group (Table 2). Among all treatment groups, Group 3 (propolis) showed minimal wound contractions on both days (Table 1). Also on day seven, the difference in wound contraction between Group 3 and the control group was not found to be statistically significant (Table 2). Although the wound contraction in Group 2 (*Hemidesmus indicus*) and Group 3 (propolis and *Hemidesmus indicus*) was significantly higher than in the control group, the difference between these two groups was not statistically significant on both days seven and 14 (Table 3).

**Table 3:** Comparison of mean scores of wound contractions in Groups 2 and 6 with each other using the multiple comparison Tukey test S: significant; NS: not significant

			Mean difference (I-J)	Standard error	p-value	95% Confidence interval	
						Lower bound	Upper bound
Day 7	Group 2	Group 3	10.68	1.37	0.0001, S	6.79	14.57
		Group 4	-2.87	1.37	0.241, NS	-6.76	1.01
		Group 5	-0.89	1.37	0.966, NS	-4.78	2.99
		Group 6	-4.81	1.37	0.008, S	-8.69	-0.92
	Group 3	Group 4	-13.55	1.37	0.0001, S	-17.44	-9.67
		Group 5	-11.57	1.37	0.0001, S	-15.46	-7.68
		Group 6	-15.49	1.37	0.0001, S	-19.38	-11.6
	Group 4	Group 5	1.98	1.37	0.607, NS	-1.9	5.87
		Group 6	-1.93	1.37	0.628, NS	-5.82	1.95
	Group 5	Group 6	-3.91	1.37	0.048, S	-7.8	-0.02
Day 14	Group 2	Group 3	3.86^*^	1.13	0.011, S	0.65	7.06
		Group 4	-3.88^*^	1.13	0.010, S	-7.08	-0.67
		Group 5	-0.8	1.13	0.953, NS	-4.01	2.39
		Group 6	-6.73	1.13	0.0001, S	-9.93	-3.53
	Group 3	Group 4	-7.74	1.13	0.0001, S	-10.94	-4.54
		Group 5	-4.67^*^	1.13	0.001, S	-7.87	-1.46
		Group 6	-10.59	1.13	0.0001, S	-13.79	-7.39
	Group 4	Group 5	3.07	1.13	0.066, NS	-0.12	6.27
		Group 6	-2.85	1.13	0.103, NS	-6.05	0.35
	Group 5	Group 6	-5.92	1.13	0.0001, S	-9.12	-2.72

The mean stress index for the treatment groups was lower in comparison to the control group on day seven as well as on day 14 (Table 4).

**Table 4:** Comparison of means of stress indices of Groups 2 to 6 with the control group (Group 1) using the Dunnett test: comparison with the control group S: significant; NS: not significant

	Group		Mean difference (I-J)	Standard error	p-value	95% Confidence interval	
						Lower bound	Upper bound
Day 1	Group 2	Group 1	-0.10371	0.46433	1.000, NS	-1.2998	1.0924
	Group 3		0.11007	0.46433	0.999, NS	-1.086	1.3062
	Group 4		-0.12436	0.46433	0.999, NS	-1.3204	1.0717
	Group 5		0.41008	0.46433	0.846, NS	-0.786	1.6062
	Group 6		-0.21186	0.46433	0.988, NS	-1.4079	0.9842
Day 2	Group 2	Group 1	-2.94057	3.85755	0.907, NS	-12.8773	6.9962
	Group 3		-4.69613	3.85755	0.622, NS	-14.6329	5.2406
	Group 4		-2.77093	3.85755	0.925, NS	-12.7077	7.1658
	Group 5		-0.31747	3.85755	1.000, NS	-10.2542	9.6193
	Group 6		-3.3471	3.85755	0.854, NS	-13.2839	6.5897
Day 7	Group 2	Group 1	-5.84589^*^	1.48082	0.001, S	-9.6604	-2.0314
	Group 3		-1.48084	1.48082	0.774, NS	-5.2953	2.3336
	Group 4		-3.65798	1.48082	0.064, NS	-7.4725	0.1565
	Group 5		-5.08458^*^	1.48082	0.005, S	-8.8991	-1.2701
	Group 6		-6.76442^*^	1.48082	0.0001, S	-10.5789	-2.9499
Day 14	Group 2	Group 1	-1.70633^*^	0.43603	0.001, S	-2.8295	-0.5832
	Group 3		-0.35392	0.43603	0.884, NS	-1.4771	0.7693
	Group 4		-0.96714	0.43603	0.114, NS	-2.0903	0.156
	Group 5		-1.25129^*^	0.43603	0.024, S	-2.3745	-0.1281
	Group 6		-2.77844^*^	0.43603	0.0001, S	-3.9016	-1.6553

**Figure 1:**
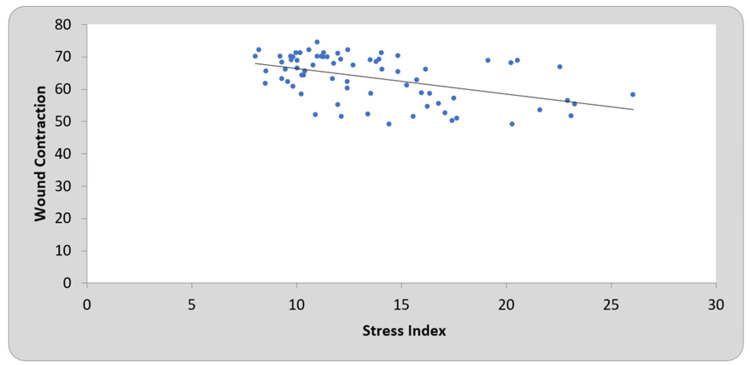
Correlation of stress index with wound contraction on day seven

**Figure 2:**
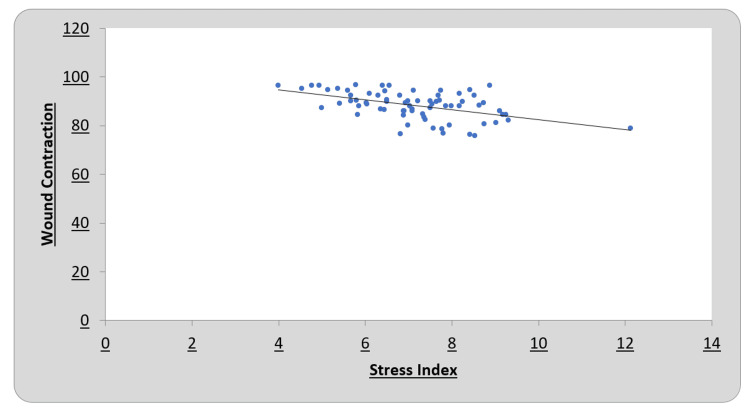
Correlation of stress index with wound contraction on day 14

A negative correlation was observed on both days between the stress index and wound contraction percentage (Figures 1-2).

## Discussion

The conduct of the research was strategized on experimental rabbits because these animal studies are not only economical but also because the relevant data thus obtained are easier to standardize and gain as compared to human studies [15]. A 5 mm punch biopsy device was used to inflict the oral ulcer. A 5 mm-sized ulcer in rabbits approximately corresponds to the size of major ulcers (more than 1 cm) in human beings.

The research was carried out to test the efficacy of different herbal wound healing and antioxidant agents and to establish a correlation between traumatic oral ulcers and oxidative stress levels in experimental animals.

Wound contraction scores

Wound contraction was measured using the method used by Bairy et al [22]. On day seven as well as on day 14, minimal wound contraction was shown by the untreated control group (Group 1), whereas maximum wound contraction was observed in Group 6 (herbal ointment and *Hemidesmus indicus* combination), followed by Group 4 (herbal ointment alone). Thus, the newly developed herbal ointment, which contains the antioxidant neem, was found to be the most effective in reducing the ulcer size in experimental rabbits. Its effect was further enhanced by the combination of systemic administrations of *Hemidesmus indicus*. The recorded parameters were on par with the observations by Pathak and Patel, where the findings suggested that herbal ointment led to significantly higher healing as compared to silver sulfadiazine on burn wounds in rabbits [16].

Among all the treatment groups, Group 3 (propolis) showed minimal wound contractions on both days. Also on day seven, the difference in wound contraction between Group 3 and the control group was not found to be statistically significant. This shows that propolis alone did not have any significant wound contraction effect on traumatic oral ulcers in rabbits. These observations are in disagreement with those made by Ali and Dahmoush, who observed that propolis had a wound-healing-enhancing effect in experimental mice [15]. This disagreement in the findings could be because of species variation, as their study was conducted in mice and they assessed wound healing at the terminal stage after the animals were sacrificed.

The wound contraction in Group 2 (*Hemidesmus indicus*) and Group 3 (propolis and *Hemidesmus indicus*) was significantly higher than in the control group, but the difference between these two groups was not statistically significant on both days seven and 14. This wound-contracting ability in these two groups can be attributed to *Hemidesmus indicus*, which is common to both groups.

Stress index scores

The oxidative stress index and protective index values obtained on day 0 served as baseline values for all the groups of rabbits, indicative of oxidative stress levels before the infliction of oral ulcers. On day one (24 hours post-wound infliction), the stress index increased in all groups. The rise in stress was similar in all groups, and no significant difference was found between them (Table 1). This indicated a sudden increase in oxidative stress levels in experimental animals after the infliction of traumatic oral ulcers.

The mean stress index of all the groups was found to decrease between days two and seven, and a further decrease was observed on day 14, which was highly significant. On day seven as well as day 14, all the groups administered with *Hemidesmus indicus *showed a significantly lower stress index (Groups 2, 5, and 6) in comparison to the control group. However, no significant difference was seen between Group 3 and Group 4 (both groups did not contain *Hemidesmus indicus*) and the untreated control group (Table 4). The maximum reduction in stress index was demonstrated by Group 6 (treated with a combination of herbal ointment and *Hemidesmus indicus*). This implies that the decline in the stress index is attributable to the antioxidant action of systemically administered *Hemidesmus indicus*, which also enhanced the healing of oral ulcers. These observations are in agreement with those of Borkar and Patel, who observed that the oral administration of *Hemidesmus indicus* in patients with pulmonary tuberculosis decreased the stress index significantly and advocated routine administration of *Hemidesmus indicus* in patients with tuberculosis [18].

To determine the correlations between the stress index and wound contraction percentage, Pearson’s coefficient was calculated on days seven and 14 for all six groups. A negative correlation was observed on both days between the stress index and wound contraction percentage (Figures 1, 2). This demonstrates that as oxidative stress decreases, wound healing progresses (as estimated by the wound contraction percentage). This demonstrates the role of oxidative stress in oral ulcers, which in turn suggests the use of systemic antioxidants for the management of oral ulcers, although confirmation by clinical trials is necessary.

Limitations

The research was undertaken on rabbits; thus, the outcomes should not be readily applied to treating human subjects. Further clinical studies are needed to do the same.

Future scope

In this study, it has been demonstrated that the administration of *Hemidesmus indicus* orally contributes to the healing of oral ulcers by decreasing oxidative stress. This may establish a definitive indication that *Hemidesmus indicus* promotes oral ulcer healing after clinical trials. The new herbal ointment used in the research has been identified as being more effective in comparison to propolis, which is currently the drug of choice for treating oral ulcers. The herbal ointment, after clinical trials, may prove to be a new, effective, and economical therapy for treating oral ulcers.

## Conclusions

A combined formulation of herbal ointment and *Hemidesmus indicus* was proven to be most effective in reducing oxidative stress and enhancing the wound contraction of oral ulcers. *Hemidesmus indicus* proved to be efficacious in reducing oxidative stress and enhancing the healing of oral ulcers. Propolis alone was not found to be effective in the promotion of wound healing or in reducing oxidative stress in experimental rabbits. A negative correlation was observed between the stress index and wound contraction scores. A combination of the new herbal ointment and *Hemidesmus indicus* after clinical trials may prove to be an effective therapy for the enhancement of the healing of traumatic oral ulcers, which may also help to reduce oxidative stress levels in the patients, although further clinical trials are required.
